# Navigating existential conversations about life and death with families in paediatric palliative care: a qualitative interview study with healthcare professionals

**DOI:** 10.1186/s12904-026-02171-4

**Published:** 2026-06-10

**Authors:** Elin Hjorth, Christina Melin-Johansson, Bodil Holmberg, Tove Godskesen, Carina Lundh Hagelin, Anneli Ozanne, Annica Lagerin, Camilla Udo

**Affiliations:** 1 Department of Health Care Sciences, Marie Cederschiöld University, Stockholm, Sweden; 2 Department of Health Care Sciences/Nursing, Mid Sweden University, Östersund, Sweden; 3 Department of Nursing Science, Sophiahemmet University, Stockholm, Sweden; 4 Department of Nursing and Health Care Sciences, Nord University, Bodø, Norway; 5 Department of Neurobiology, Care Sciences and Society, Karolinska Institutet, Stockholm, Sweden; 6 Institute of Health and Care Sciences, Gothenburg University, Gothenburg, Sweden; 7 Sahlgrenska University Hospital, Gothenburg, Sweden; 8 Department of Health and Welfare, Dalarna University, Falun, Sweden

**Keywords:** Communication, Existential conversations, Family, Healthcare professionals, Paediatric palliative care

## Abstract

**Background:**

When a child has a life-threatening illness, guidelines emphasise the importance of providing holistic support to the whole family. Conversations about existential issues are essential in this support, yet staff often lack formal training in communication, particularly when addressing such issues as life and death. This qualitative study explored how paediatric healthcare professionals in Sweden experience and approachconversations about existential issues with children and their families facing life-threatening illness.

**Methods:**

Eight professionals representing different disciplines and paediatric palliative care settings took part in individual interviews, analysed using an interpretative description approach.

**Results:**

The results show that healthcare professionals experienced the existential conversations with families as emotionally intense, ethically complex, yet meaningful. A balance between courage, presence, sensitivity, and professional boundaries was crucial, as well as the need for collegial support. The importance of honest communication and age-appropriate ways of talking about illness and death was emphasized. They stressed that communication should be empathetic and culturally sensitive, without ready-made solutions, and based on the unique needs of the family. They also described organizational factors that influenced their ability to engage in existential conversations with the families.

**Conclusions:**

Maintaining an open approach to existential conversations is essential to how these dialogues are carried out. Such an approach includes creating workplaces with an organizational culture that encourages existential conversations with patients and families. The importance of both personal and professional development is also emphasized, where collegial support and structures that promote professional growth, such as supervision and debriefing, strengthen the ability to address existential issues.

## Introduction

Conversations about death and serious illness, particularly in relation to seriously ill children, remain a significant challenge for healthcare professionals (HCPs) [1, 2]. While hope for survival often persists until death, it is essential that HCPs who care for and support the child and family address the severity of the illness early in the disease trajectory [3]. Confidential and existential conversations occur as a mutual process between patients and HCP which is both delicate and requires continuous presence and sensitivity. These conversations can be initiated by either the patient or the staff and take place in an interaction where trust between the parties is essential [4]. To be able to conduct these conversations, certain skills are required, for example prior reflection on caring situations from an existential perspective (contributing to understanding and emotional preparedness to remain present) [5]. Further, it is crucial that HCPs build trust, and show respect and empathy in conversations, and also respect parents as experts about their child [6].

This study focuses on children with life-threatening illnesses and their families. A life-threatening condition is defined as one with a high probability of premature death due to severe illness, yet also a possibility of long-term survival into adulthood. Examples include cancer, severe neurological impairments, acute injuries requiring intensive care and conditions giving rise to a dependence on technology [3].

According to the World Health Organization [7], paediatric palliative care should provide holistic care, encompassing the child’s physical, psychological, and spiritual dimensions, while also offering support to the family. Existential questions frequently arise in palliative care when families face profoundly challenging circumstances. These questions often relate to themes such as meaning, loneliness, mortality, and freedom, and are considered universal across cultures, genders, and religions [8]. Issues concerning meaning, life and death, and how to find strength are often present in care situations, particularly when meeting a person with a life-threatening illness [9]. Within palliative care, it has been emphasised that spiritual and existential suffering must be systematically identified and addressed, and that interventions should, as far as possible, be grounded in evidence and established best practices [10].

To address the holistic needs of children with life-threatening illnesses, effective communication between HCPs and families is essential. Such communication ensures that critical information is conveyed, while also allowing the wishes and priorities of both the child and family to be understood, thereby enabling shared decision-making on important issues. As stated in Article 12 of the United Nations Convention on the Rights of the Child (UNCRC), children have the right to express their views, receive relevant information, and participate in decisions that affect them [11]. Contrary to common parental assumptions, research indicates that children with palliative care needs are often aware of their situation and wish to be informed about their illness [1, 12]. Withholding information may lead children to imagine scenarios worse than reality, and many of them attempt to protect their loved ones from their own distress – a phenomenon documented for decades [13].

While conversations are crucial in palliative care, discussing life when close to death, or when death is impending, is a complex task. Views of children within paediatric healthcare have changed significantly over time, and children are now recognised as being aware of death having the right to receive individually and age-adapted honest information and thus participation in care decisions [14, 15]. However, in conversations, this requires careful consideration and a balance between values, the child’s developmental level, parents’ wishes and cultural norms, all of which need to be taken into account [15]. Prior research suggests that conversations with families about a child’s terminal illness require preparation, experience, and training, which is often lacking [16, 17]. Despite growing evidence supporting the benefits of open communication in end-of-life care, HCPs often struggle to talk about death with the child and family, and when words are difficult to find, silence and other forms of non-verbal communication may take the place of spoken conversation [2]. Communication training aimed at strengthening communication among HCPs working with children with life‑threatening conditions and their families do exist, but it remains a relatively limited area [18].

Further, to support children with life-threatening illnesses, the primary focus is on maintaining their everyday lives. To safeguard the child’s right to play, rest, and leisure, a wide range of activities have been integrated into Swedish paediatric palliative care, including play therapy, music therapy, hospital clowns, hospital schooling, and creative activities. Clown interventions are an integrated component of Swedish paediatric care and are also used internationally as an effective means of alleviating the negative emotions among hospitalized children [19]. Despite the existence of national guidelines, paediatric palliative care in Sweden is organised differently across regions, largely due to the country’s decentralised healthcare system [20]. Consequently, access to paediatric palliative care varies significantly, with some regions providing dedicated paediatric palliative care, whereas others rely on adult palliative care units or general inpatient services for children, including hospice care primarily designed for adults [21].

As stated above, it is central to talk about life, dying, and death when supporting children with life-threatening illness and their families. However, how HCPs experience and approach existential conversations about these topics when a child has a life-threatening illness has yet to be fully explored. Gaining knowledge about who initiates these conversations, how they are conducted, and what issues are addressed, and in what way, may help guide HCPs in these conversations.

### Aim

The study explores how HCPs in Sweden experience and approach conversations about existential issues with children and their families facing life-threatening illness.

## Materials and methods

### Design

This qualitative study forms part of a larger, mixed-method research project, *Talk for life – conversations in palliative care (LIFE-Talk).* The project investigates how HCPs in Sweden experience conversations about existential issues that address life-course aspects with patients and their families in different palliative care contexts, including care of both children and adults.

Data for this study were collected between November 2023 and September 2024 through individual interviews with HCPs working with children in need of palliative care and their families. The study adopted an inductive, qualitative design using interpretive description [22]. This method of analysis was chosen for its exploratory nature, which allows for an in-depth examination of HCPs’ experiences and ways of conducting conversations about existential issues. The Consolidated Criteria for Reporting Qualitative Research (COREQ) checklist was used for this study [23].

### Settings, recruitment, and participants

The study was performed in paediatric healthcare settings providing care for children (aged 0–18) with life-threatening illness. These included general children’s hospitals, specialised paediatric palliative care units, and a specialised neurology unit. Participants were recruited from different paediatric healthcare settings in central Sweden, representing both urban and rural contexts. One additional unit in northern Sweden was invited to participate but declined due to heavy workloads.

A strategic sampling approach was employed. Initially, unit managers were contacted, who then facilitated contact with HCPs interested in participating. HCPs received both written and oral information about the study. Inclusion criteria required the participants to have at least six months of experience working with children facing life-threatening illness.

In total, eight HCPs consented to participate, including registered nurses (*n* = 3), social workers (*n* = 2), a physician (*n* = 1), a hospital clown (*n* = 1), and a hospital teacher (*n* = 1). All participants were women, born in Sweden and spoke Swedish. They were between the ages of 35–55 and had worked at their respective unit 6 years or longer. Five participants held additional education in palliative care.

In two cases, there was a prior acquaintance between the researcher and the participants. These relationships were limited to professional contexts and did not involve close personal ties. To address the potential influence of this familiarity, all interviews were anonymized and coded prior to analysis. Throughout the analytic process, the researchers maintained an explicit awareness of the risk of preconceptions and consciously avoided drawing on prior knowledge of the participants, approaching the material in a neutral and systematic manner.

### Data collection

A digital questionnaire was used to collect demographic and clinical information before the individual interviews. A semi-structured interview guide with open and follow-up questions was used, designed by the first, second, and last authors. The interviews began with an open-ended question: ‘*Can you tell me how you talk to families of children with life-threatening illness?*’ The interview questions were structured around three domains (see Table 1). While maintaining a logical flow, the guide was used with flexibility to allow for sufficient sensitivity with which to capture the participants’ narrative. The interviews aimed to explore conversations about existential issues, but since the concept can be perceived as abstract and difficult to understand, questions were also asked about ‘deeper’ conversations as described in Table 1. The interviews were audio-recorded and held via digital video calls. They lasted between 40 and 90 min (md = 43). The interviews were performed by the first and last author.

**Table 1:** Semi-structured interview guide

Domain 1: The conduct of existential conversations with families
Can you tell me how you talk to families of children with life-threatening illnesses?
When are in-depth conversations, for example, about existential issues, with the family conducted in palliative care?
When you talk with the family, who is included?
What questions are raised by the ill children, and their sibling and parents, during the conversations?
How are these conversations adapted to the family’s needs?
How can families be supported when experiencing, for instance, feelings of helplessness, frustration, and lack of control?
How do you talk about the future?
Domain 2: The individual’s personal experience of these conversations
Can you describe a conversation that has strongly affected you?
What thoughts and feelings arise for you?
Domain 3: Ways in which conversations are approached at a collegial group level
How do you and your colleagues talk about these conversations with families?
What support do you need in relation to conducting in-depth conversations with families?

### Data analysis

The data analysis was conducted according to the principles described by Thorne [22] to generate clinically relevant knowledge. The audio-recorded interviews were transcribed with the assistance of transcription software in a text-processing program and then reviewed to ensure their accuracy. Meaning units were identified and coded by the first author, with particular attention given to parts that stood out or aroused curiosity in relation to the research aim. The first author organised the themes into a coherent narrative, which was then discussed with the second and last authors. Guided by Thorne’s approach, the analysis focused on remaining true to the study’s aim and identifying relevant patterns in the data by continuously asking, ‘What would this mean in applied practice?’

The description that emerged was divided into meaningful themes related to HCPs’ experiences of conversations with children and families in palliative care. To showcase that the themes were grounded in data, the results were illustrated with verbatim quotes. Continued discussions among the authors focused on comparing interpretations, resolving discrepancies in coding, and refining emerging themes, which further advanced the analytic process. The analysis was considered complete when the first, second, and last authors agreed on the results.

## Results

**Fig. 1:**
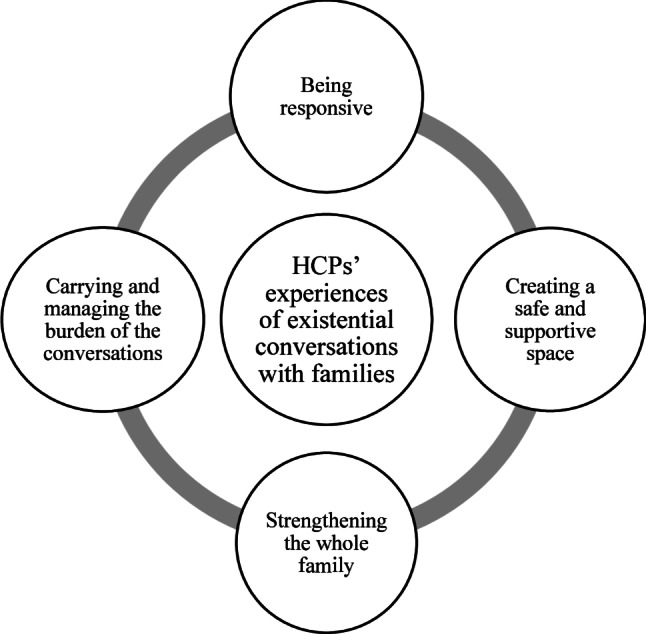
Overview of themes

The analysis resulted in four themes: *Being responsive*; *Creating a safe and supportive space*; *Strengthening the whole family*; and *Carrying and managing the burden of the conversations* (see Fig. 1).

### Being responsive

The HCPs emphasised the importance of being responsive in encounters with the children and their families in order to fully account for their priorities and concerns. The HCPs described the initial contact with families as often acute, such as at the diagnosis of a life-threatening illness or in a critical phase of the illness. In these moments of profound vulnerability, HCPs described being deeply affected and stressed the importance of showing respect and attentive listening, sometimes providing concrete suggestions to guide the family through continuous dialogue. The HCPs described balancing medical information with listening and compassionate care. Clear communication using concrete words was described as central when supporting families in complex existential situations and decision-making about their child’s care or after the child’s death. For the HCPs, being responsive meant adapting conversations in shifting circumstances (e.g., sudden changes in a child’s condition), preserving and supporting the family’s sense of agency and emotional safety, while also offering concrete guidance based on their professional experience. As one HCP described:

I try to first listen and take in the parents’ perspective. Then, I come up with my own reflections and my experience as a nurse. (HCP 1)

A recurring topic was addressing death in an open and honest manner. HCPs expressed their need to reflect on existential issues in order to enable such conversations, as well as having the courage to raise and explore questions about death as a possible outcome for the ill child. Rather than avoiding or shying away from the difficult subject, facing the issue head-on could help families understand and process the situation in their own pace.

The uncertainty is almost always great and painful. You can say, in general, that not knowing is something that torments people, and it creates room for fantasies. So that’s why I’m not afraid to talk about death, because what I mean by that is that we can talk about all the potential outcomes, and it’s my job as a representative of medical care to be as prepared as I can for all the potential things that can happen to the child. (HCP 2)

Being responsive to each family’s cultural and spiritual values was highlighted as crucial in both practical and existential conversations. HCPs noted that cultural background influenced how families cope with illness and death. In some cases, families experience shame or denial related to the child’s illness, which was perceived as challenging when the child understood their situation and asked questions. The HCPs described how conversations with children and their families about the future and death must be shaped by the family’s unique needs. Such conversations require sensitivity, empathy, and an ability to adapt to each person’s wishes and emotional state, as well as managing one’s own reactions. The HCPs repeatedly cited the importance of timing in these matters, emphasising that one must meet the person where they are and not force sensitive topics on them. As one HCP described:

I have decided that I should not be the one to initiate [conversations about the future and death], though I may ask sometimes. But when it comes up, I always take the opportunity to listen. It’s very important to meet them where they are and listen. Sometimes, just listening is enough. (HCP 3)

However, the HCPs noted that pausing to ‘catch the moment’ for such talks was often difficult due to time constraints. Indeed, they described how a pressured schedule often prevents them from having as much time for conversations as they would like. This can make it difficult to build the trust needed for the child to feel comfortable talking. At the same time, some noted that the lack of time can also feel like a relief, as it would spare them from becoming emotionally involved in every relationship.

Children in palliative care were described as often living in the present and focusing on experiencing joy rather than illness or impending death. HCPs aimed to help children fulfil their wishes, sometimes with the awareness (but without explicitly stating) that it might be the last time the child would engage in a particular activity. However, some (especially older) children were described as able to express specific thoughts about their own death, such as planning their funeral or reflecting on their family’s future. HCPs described these existential conversations as emotionally difficult, but emphasised their commitment to remain present for the child’s sake. One HCP shared:

I’m working with a child, who is very communicative. She verbalises what she thinks and feels. I think the conversations are bloody difficult sometimes. […] It’s difficult because there is always, at least in me, a desire to comfort or rescue or do something good, give an ice cream or whatever you want. And when I sit with a child who is dying and who talks to me about how she should prepare for her death, asking, ‘How should I prepare? What is it like to be dead? I’m just scared.’ […] It’s not an easy job. You can’t simplify it and you need courage, knowledge and support. (HCP 6)

### Creating a safe and supportive space

To enhance the child’s sense of security, conversations would often be held in their parents’ presence. HCPs described how they try to adapt to the child’s wishes when planning a conversation. This could involve preparing the child by explaining the purpose and framework of the discussion, allowing them to decide how it should be conducted, and choosing whether to participate themselves or let the parents have the conversation alone. Across professional roles, HCPs aimed to promote spaces where both children and parents could openly express their thoughts, emotions, and concerns. Building trust was seen as the foundation for deeper discussions, including both existential and practical matters related to death. One HCP described a situation where a child was critically ill:

We made hand- and footprints with the child to create memories together. You create a safe atmosphere with the parents in various ways. In that safe space, I could then say, ‘I would like to talk more about what happens when a child dies on the ward, are you ready to talk about that?’ It was like opening a door. They responded, ‘Oh, we’ve been thinking about this a lot, we are really ready’. (HCP 4)

Conversations could often occur in everyday settings, such as in patient kitchens or in the corridor, where parents would open up and spontaneously initiate discussions about practical, emotional, and existential issues. The HCPs noted that adults were more likely to engage in structured conversations than children. Conversations with a child could instead involve joking and playing. These less formal moments were described as vital for forming deeper connections.

The conversations might seem trivial or about silly things, but I think they can still touch us deeply, both for us, as professionals, and them, as families. It’s about reaching each other, but in a different way. (HCP 5)

Having met the family before and being truly present – that is, listening actively and attentively when parents or children spontaneously raised existential issues – was described as essential for making them feel safe enough to share their thoughts when they were ready to do so. Relationships with families living with chronic conditions were described as often evolving over time, enabling profound discussions about the future and death.

### Strengthening the whole family

HCPs emphasised the provision of holistic support to the entire family by helping them to communicate openly with one another, process difficult emotions, and find age-appropriate ways to cope with illness, death, and loss. Some parents aiming to protect their child from distress would avoid morbid issues with their child. The HCPs perceived this as potentially limiting the child’s ability to understand and process their situation. Accordingly, they emphasised the importance of supporting families in establishing open and honest communication tailored to the child’s age and needs. Creative tools, such as reading, drawing, or painting, were occasionally used to make such existential conversations more accessible. The HCPs described how they worked to support parents in initiating these conversations on the child’s terms, thus ensuring that the child’s voice was heard and that their preferences for receiving information were respected. One HCP described how she would provide families with practical tools and strategies to support open communication about emotions:

The children often come up with their own concrete suggestions, such as talking after brushing their teeth or using tools like an emotion thermometer to show how they are feeling. This tends to work well in helping families communicate better, and most parents are responsive to their children’s needs and preferences about how the conversations should take place. (HCP 6)

Supporting siblings was considered an important aspect of paediatric palliative care. This could involve helping siblings understand and process situations in age-appropriate ways, or facilitating parents’ decision-making over whether siblings should visit their ill brother or sister. It was also considered important to give siblings the opportunity to share their concerns with HCPs. One HCP recounted how a child, during a short walk, found the courage to ask deeply existential questions about their sibling’s death and the future of their soon-to-be bereaved family. HCPs emphasised the importance of recognising such opportunities and using them to support siblings.

Clear, concrete language about death was considered essential, avoiding such euphemisms as ‘going to sleep’ to prevent misunderstandings. When informing siblings about an impending loss, honesty and reassurance were seen as important. An HCP described occasionally needing to help parents inform a sibling about an impending death:

It’s not like I can go in and sit with siblings and start informing them without having their parents’ OK. […] It’s not like the sibling is sitting on a chair and I’m sitting on a chair with a table. No, that’s not how you talk to a five-year-old. You sit on the floor or in bed and maybe play with a cuddly toy and talk, then you ask, ‘do you know why you’re here?’ and you get, ‘yes, he’s ill, then we’re going home’. Then you have to say, ‘you’ll go home without him’. You have to try to be clear because otherwise it’ll just be rubbish. […] Later you can pick it up again. ‘Do you remember what we talked about? Have you thought any more about it? Should we talk to mum and dad?’ (HCP 7).

Further to traditional conversations, creative activities, such as those found in playtime, were valued for offering siblings both emotional respite and openings for spontaneous, low-pressure discussions. HCPs also stressed supporting people outside the immediate family, such as schoolteachers, by providing guidance on how to approach conversations with peers about the situation. A challenge described was parents’ difficulty in sharing their child’s illness with others. HCPs emphasised that helping parents receive support from extended family and friends can reduce the isolation they feel during these challenging times. One HCP shared:

I try to support the family by encouraging them to think that if there’s ever a time to rely on their network, it’s now. And it’s also a benefit for the child, as new people bring in new energy. (HCP 2)

They also emphasised giving parents opportunities to talk privately and find peer support without interruption from their children or other family members. This can help parents process their feelings, formulate questions, and prepare for further conversations with the child and the rest of the family.

### Carrying and managing the burden of the conversations

The HCPs stressed the importance of grounding oneself by, for example, reflecting on existential issues and their own spiritual positions, and being open and able to connect with others on such issues. Being emotionally available and attuned to the family’s needs was considered more important than finding the ‘right’ words. Over time, HCPs described how their focus had shifted from their own role to prioritising the family’s experience and needs. One HCP reflected:

At the beginning of my career, I did not understand the importance of involving the whole family and saw my own role as central. Now I see how important it is for each individual to express their concerns and needs. (HCP 6)

Meetings with families were described as challenging, but often deeply rewarding in that a real contribution could be made to the family’s lives. Balancing professional and personal involvement was emphasised as essential for maintaining sustainable relationships, especially when contact continued after a child’s death. Such contact calls for a delicate balance between professional closeness and personal boundaries. At the same time, HCPs emphasised that strong relationships often enabled families to open up about death, grief, and existential uncertainty.

You become an important pillar in the family. Then it’s difficult when the child has died. I have some parents who have continued to contact me. And that’s where it’s difficult. I have to draw a line. For example, I’ve said that you can’t use my private mobile phone. But they feel that we are their family, the only ones who know what’s happened and who have followed this. (HCP 3)

While training in conversations was valued, personal experience and collegial reflection were seen as key to the continued development of the emotional tools needed when caring for and supporting the child and family. Indeed, collegial support and teamwork were described as essential buffers against emotional overload. Informal handovers and check-ins were valued for sharing clinical information and ensuring continuity of care and emotional coherence within the team. A supportive workplace culture, allowing space for emotional expression and humour, was also highly appraised. While occasionally described as lacking, structured supervision was seen as valuable for helping one reflect on complex cases and find shared meaning. One HCP described the culture within their team:

It’s very open. You can even make jokes about difficult things in a way that might be perceived as insensitive by an outsider. But you need a certain amount of humour to keep you going. But they [colleagues] are also very warm, and they give me confirmation of what support I have provided for the family. We can also be physical with each other. We can hug, and we can cry and show emotions to each other. It’s an incredible [source of] support. (HCP 1)

The HCPs also mentioned that barriers in the workplace culture can hinder conversations about existential issues. For instance, fear of ‘saying the wrong thing’ or causing distress would sometimes lead to avoiding discussions about death. One HCP remarked:

There are still many [colleagues] who believe that children shouldn’t be talked to about it [death], because they think it will upset the child. […] Even though staff are trained, the idea that you shouldn’t talk about it is still quite strong. And it comes up fairly often. And I think that has actually been surprising. It’s as if people’s opinions or fears carry more weight than the knowledge we have. They think their own beliefs are more important than what we actually know. (HCP 6)

Finally, HCPs reflected on their roles within existential conversations. They described how some families form bonds with specific HCPs, and that the person who becomes most important can vary from family to family. They also stressed how it is not the individual themselves that matters most, but the professional role they represent. Although deep conversations with families can be challenging, the HCPs reported how these meetings and relationships contribute to a profound sense of meaning in their professional practice.

There’s this idea that the job is inherently demanding – which it is – but, at the same time, it’s not only that. Because it’s also… life-giving. It gives me so much love. And there’s love in being given the opportunity to have conversations with families that may be difficult. It’s like walking on glass sometimes, because you’ve been entrusted with something. And to me, that trust is… a blessing. It’s something I feel I need to care for, to hold gently. That’s what I think is at the heart of it all. (HCP 1)

## Discussion

This study sought to explore how paediatric HCPs experience and conduct conversations about existential issues with children facing life-threatening illnesses and their families. The following discussion centres on the importance of establishing a clear and honest communication with and within the family, as well as the significance of professional support.

The participating HCPs viewed it as part of their role to create conditions for conversations in which each family member could express themselves, both with professionals and within the family. Previous studies have shown that parents need various forms of support for discussing end-of-life issues with their child, and have highlighted the importance of professionals offering support to prepare for such conversations [24, 25]. Moreover, and as emphasised in the interviews, professionals must remain sensitive to family preferences and respect the extent to which they wish HCPs to be involved in the care of their child and decision making. These existential conversations are challenging, particularly when parents and HCPs hold different views of death and how it can be discussed [1, 17], which underscores the need for an individualised approach.

The participants emphasised the value of offering children honest information about an approaching death when they showed a desire to talk, describing openness as a way to reduce fear and uncertainty. Parents are often torn between striving to prolong their child’s life and wanting to protect them from suffering. In some cases, this leads to withholding information, with many parents preferring not to talk about death with their ill child [26]. And yet, research shows that age-appropriate, sensitive communication benefits both the child and the family [25]. In line with previous research, clarity was considered a critical component of communication, complementing sensitivity and regarded as a form of care in itself [27]. However, studies centring on bereaved parents have highlighted persistent challenges, such as providing information about incurability and impending death too late, thus leaving families unprepared and deprived of opportunities for meaningful goodbyes [28]. This reinforces the importance of timely and sensitive conversations about death. Although communication often occurred primarily through parents, several of the interviewed HCPs emphasised the importance of including children according to their age, maturity, and willingness. A systematic review has highlighted that paediatric end-of-life communication can be improved both between parents and children and between families and healthcare teams, noting that parents may benefit from guidance on what information can and should be shared with their sick child and its siblings [26].

Professional development and how HCPs can develop competence in communication with families were both highlighted as topics. Indeed, the participants noted that they did not consider formal training to be a crucial factor in their ability to engage in existential conversations, but rather stressed the need for reflection and awareness on existential issues and their effects. Yamaji et al. [18] conducted a systematic review examining the impact of communication skills training programs for HCPs caring for children with life-threatening conditions. The authors found no evidence that such programs improve the quality of life or satisfaction of patients and their families. However, the review indicates the training may positively influence HCPs’ attitudes toward communication. Furthermore, a separate review article shows that simulation‑based training can support a sense of personal development among nurses pursuing advanced education in palliative care, although findings remain contradictory regarding whether such training increases students’ confidence in their communication skills [29]. Building on these studies [18, 29], communication skills training for HCPs may help foster a positive organisational culture by extending beyond technical communication strategies to include methods that support reflection and strengthen attitudes toward difficult conversations. This also aligns with the present study, which found that collegial support and opportunities for shared reflection both informally among colleagues and through structured interventions such as Schwartz Rounds, may enhance professional competence and help protect against emotional overload [30]. Taylor et al. [31] reported that healthcare staff perceived Schwartz Center Rounds as valuable for reflection and support in managing the emotional challenges of clinical work, while also noting that the overall evidence base remains limited.

HCPs described that close relationships with children and their families could affect them both personally and professionally. The participants emphasised the importance of balancing professional and personal involvement to maintain sustainable relationships. At the same time, engagement with patients was described as contributing to a sense of meaning and job satisfaction. The HCPs highlighted the need to remain grounded in their own existential reflections and spiritual perspectives, suggesting that self-awareness and personal resilience are essential for managing emotionally challenging situations. Setting boundaries between work and private life, experiencing one’s work as meaningful, and having supportive and trusting relationships with colleagues and management have previously been described as important self-care strategies for preventing professional burnout among paediatric healthcare professionals [32]. The participants identified supervision and debriefing as valuable forms of support, although such structures were not always formally established in their workplaces. Previous research has uncovered mixed perspectives regarding these support forms: While some professionals found supervision and debriefing helpful and wished it were more regular, others did not perceive it as particularly beneficial [33]. These findings indicate a need for more flexible and individualised support structures tailored to the specific needs of HCPs. Based on the present findings, one proposal is to clarify, for example in the National Clinical Guideline for Paediatric Palliative Care [20], how honest, age-appropriate communication about illness and death should be conducted in an empathetic and culturally sensitive manner. It could also outline how organizational factors, such as a workplace culture that encourages existential conversations with patients and families, influence the ability to engage in such conversations and thereby create better conditions for them.

### Strength and limitations

This study makes a unique contribution by exploring the experiences of HCPs from five different disciplines working in diverse clinical settings within paediatric palliative care. The physician, hospital social workers, registered nurses, hospital teacher, and hospital clown generously shared both personal and professional insights from their work with children facing the end of their lives. Recruiting participants proved challenging due to organisational barriers, such as time constraints. Nevertheless, despite the relatively small number of participants, the dataset was rich, and recurring themes emerged across different perspectives, which were deemed sufficient for the study.

The participants were highly engaged in the topic; some were noticeably emotionally affected during the interviews, indicating that participation involved a potential risk of emotional discomfort or distress. At the same time, the interviews also provided an opportunity for participants to reflect on the subject. Including participants from different professional backgrounds should be considered a strength of the study, as it highlighted how various roles contribute different perspectives on conversations about life and death with families. Hospital teachers and hospital clowns are professional groups that often work closely with families in paediatric palliative care, yet they are rarely represented in scientific research. Although each encounter and its purpose differ depending on the profession involved, existential philosophy [34–36] suggests that addressing existential issues is primarily a human encounter requiring not only theoretical knowledge, but above all, personal authenticity.

However, a notable limitation of the study is the absence of assistant nurses among the HCPs. In Swedish paediatric palliative care, assistant nurses often have close and continuous contact with children and their families and may be involved in both practical and emotionally meaningful conversations. In addition, other professionals who are commonly part of the multidisciplinary team, such as psychologists, physiotherapists, occupational therapist and dietitians, were not included. The lack of representation from these professional groups may have resulted in the omission of perspectives that could have enriched the understanding of conversations about existential issues.

The study’s trustworthiness was strengthened through a systematic analysis process in which authors read the material, compared interpretations, and discussed discrepancies. This approach enhanced the credibility and confirmability of the findings. The extensive use of quotations further illustrates how the interpretations are grounded in the data. Transferability is left to the reader’s judgement, although the description of the study context is intended to support such an assessment. However, it is important to acknowledge that the topic is culturally sensitive, and the findings are unlikely to reflect all cultural perspectives on death.

## Conclusion

The results underscore the importance of HCPs being open and prepared for conversations about existential issues when they arise. Building trusting relationships and promote a sense of security by being present and responsive when parents, ill children, or their siblings wish to engage in these conversations is essential. Furthermore, the results indicate that, although discussions about life and death with families can be challenging for HCPs, they are nevertheless perceived as a meaningful and rewarding aspect of professional practice. Offering collegial support and structures that promote professional development (which the participants regarded as valuable resources) would be beneficial to managing these emotionally and existentially demanding conversations. Personal and professional development initiatives e.g., supervision and debriefing, that strengthen the ability to address existential issues, alongside efforts to foster a positive organisational culture that encourages engagement in existential dialogue, should be prioritised by management. Such measures may have the potential to promote openness and interest in conversations with families and may contribute to creating conditions that enable HCP to prioritise and navigate existential discussions about life and death in paediatric palliative care.

## Data Availability

Due to privacy regulations, the datasets generated and/or analysed during the current study are not publicly available but are available from the corresponding author upon reasonable request.
